# Heat Analysis of Thermal Conductive Polymer Composites: Reference Temperature History in Pure Polymer Matrices

**DOI:** 10.3390/polym14102084

**Published:** 2022-05-20

**Authors:** Fethi Guesmi, Makram Elfarhani, Ali Mkaddem, Sami Ghazali, Abdullah S. Bin Mahfouz, Abdessalem Jarraya

**Affiliations:** 1 LA2MP, National School of Engineering of Sfax, University of Sfax, P.O. Box 1173, Sfax 3038, Tunisia; fethiguesmi2017@gmail.com (F.G.); makram.farhani@gmail.com (M.E.); ajarraya@uj.edu.sa (A.J.); 2 Department of Mechanical and Materials Engineering, FOE, University of Jeddah, P.O. Box 80327, Jeddah 21589, Saudi Arabia; sghazali@uj.edu.sa; 3 Department of Chemical Engineering, FOE, University of Jeddah, P.O. Box 80327, Jeddah 21589, Saudi Arabia; asbinmahfouz@uj.edu.sa

**Keywords:** TCP, glass fiber, epoxy, polyester, temperature, milling, SEM

## Abstract

This attempt aims at assessing heat generation in thermal conductive polymer (TCP) composites widely used in aerospace sectors. Temperature histories were investigated in both nonreinforced and glass-fiber-reinforced TCPs during abrasive milling. Glass/epoxy and glass/polyester composites with 30% unidirectional glass fiber content were prepared according to appropriate curing cycles. Type K thermocouples connected to a data acquisition system ensured the recording of temperature history along the trim plan during milling. Unexpectedly, when milling TCP composites parallel to fibers, peak temperature was found to be slightly lower than that recorded in nonreinforced polymers. The lateral surface of fibers acts to favor sliding friction, which limits heat generation at interfaces, while relatively low specific heat capacity and thermal conductivity of glass fiber disadvantage heat transfer. However, when milling perpendicular to fibers, the contact area between the tool and the transverse failure area of fibers increases drastically, hence involving severe friction at interfaces. This yields peak temperatures sensitively higher than those obtained in nonreinforced polymers. SEM inspections highlighted the failure modes dominating the material removal process in both nonreinforced and glass-fiber-reinforced polymers. The microcracks and debris observed at the trim plan explain, in part, the heat generation detected on temperature rate plots. Thus, heat conduction between phases governs sensitive surface finish integrity and tool lifetime and, hence, has great economic impact on the manufacturing steps.

## 1. Introduction

Fiber-reinforced thermal conductive polymers (TCPs) have become increasingly popular in several industrial applications because of their attractive specific properties. Especially, they offer good thermal and chemical resistance and high specific strengths. In addition, these materials present many advantages in manufacturing, as reported by Zitoune et al. [1]. These reasons make them serious candidates for substituting conventional metals in the automotive, aeronautic, and optics sectors [2,3]. Although the molding processes have proved their reliability in providing net-shape products, machining remains highly required to ensure efficient assembling in many cases [4,5,6]. However, when compared with conventional metals, TCP composites exhibit various issues in machining. On the one hand, the orthotropic structures and heterogeneous phases resulting in significant disparity in properties of the fiber and matrix make it difficult to control the damage mechanisms acting alone or in close interaction during machining, as typically reported by Mohan et al. [7] and Karatas et al. [8]. On the other hand, the fiber abrasiveness complicates the tool selection and affects sensitively the tool lifetime. Indeed, these materials are made by the combination of two main synergistic constituents, namely, reinforcement and matrix, to improve property-to-density ratios. Caggiano [9] highlighted that the matrix and reinforcement types decide the composite structure characteristics. Fiber-reinforced thermoset polymer laminates are basically classified into two types: CFRP (carbon-fiber-reinforced polymer) and GFRP (glass-fiber-reinforced polymer). A large number of researchers have discussed the challenges encountered in machining composite materials by addressing purely mechanical approaches. Pure thermal or thermomechanical effects have been found to be understudied during heat generation when machining seems to play a key role in the material removal process.

Controlling the process conditions has been the key topic addressed since the first attempts of composite cutting. Several investigations have been carried out to highlight the influence of cutting parameters, such as cutting speed, feed rate, and depth of cut on the output variables (e.g., surface quality, tool performance, forces, and generated heat). Typically, Davim et al. [10,11] performed milling tests on polymer composites and concluded that feed rate is the main parameter influencing the machining force. Furthermore, investigations by Zenia et al. [12] led them to deduce that the cutting force increases as well with the depth of the cut. Authors have indicated that the former parameter affects also the induced damage levels. In the same context, Wang et al. [13] developed an ANOVA mathematical model to statistically analyze the influences of milling parameters on the surface roughness of CFRP composites. They pointed out that combining the minimum feed rate and medium spindle speed with the minimum radial depth of the cut clearly improves the cut surface quality. Based on experimental tests, Abhishek et al. [14] proposed a hybrid optimization methodology combining genetic and ANFIS algorithms to optimize the depth of the cut, feed rate, and spindle speed for drilling GFRP.

In fact, most of the experimental studies that have been conducted to find out the optimum machining parameters have the ultimate goal of avoiding critical failures, such as matrix–fiber debonding, microcrack formation, stress concentration, and particularly delamination within the fresh surface during edge milling of GFRP and CFRP materials [8,15,16]. Indeed, the quality of the machined surface is one of the substantial concerns to be considered when deciding about the composite structure integrity. While inspecting milled parts, Hocheng et al. [15] announced that the quality of the surface finish distinctly depends on the fiber orientation. More accurately, the best surface qualities were obtained when the fibers were parallel to the feed tool direction, instead of having perpendicular orientation, which gives rise to more severe failures. Based on SEM analyses, Pecat et al. [16] evaluated the quality of milled surfaces in four different fiber orientations. It was found that a smooth surface was reached for milling at 0° and +45°, while micrographs obtained at −45° and 90° illustrated serious damage involving microcracks and segmentations. The authors attributed these damage features to the variation of cutting mechanisms in each fiber orientation case. Karpat et al. [17] pointed out that adjusting the fiber orientation at 45° improves the surface quality in milling unidirectional CFRP laminates. Hintze et al. [18] specified that tool wear is a major factor of delamination during CFRP slot milling. While investigating GFRP milling, Erkan et al. [19] observed that the damage level decreases sensitively with the increase in the tool number of flutes.

Actually, many other authors insist that tool performance is the key issue that needs to be focused on to increase efficiency in cutting TCP composites. Faria et al. [20] compared the wear resistances of two tool-grade materials after drilling GFRP laminates. The authors established that compared with the high-speed steel drill, the cemented carbide one manifested lower wear land. These results were confirmed by Kavad et al. [21], who mentioned that harder tool materials likewise reduced the delamination effects in processing GFRP. While conducting a review, Lokesh et al. [22] found a correlation between cutting conditions and tool wear. Based on analysis of the variance method of the collected experimented data, the authors deduced that combining relatively low feed and high cutting speed improves tool life. Criado et al. [23] analyzed the influence of tool geometry on the damage involved in a thick multidirectional CFRP laminate during the orthogonal cutting process. From experiments, it was observed that a more robust geometry, with a lower rake angle and a higher cutting-edge radius, favors the thrust force rise, hence resulting in delamination and burrs. Sheikh-Ahmad et al. [24] pointed out that the rise of the cutting tool temperature causes drops in its hardness and consequently accelerates tool wear. Indeed, besides the tool life and the final composite structure quality, increase in cutting temperatures during the machining process can significantly affect the cutting mechanisms themselves, and therefore, the adjustment range of cutting parameters. Hence, the assessment of heat partition when cutting TCP composites is a challenging task that has been recently addressed by several research studies. Understanding the sensitive interaction between thermomechanical elementary mechanisms should provide useful information to control the material removal process, as proved by Sorrentino et al. [25]. In addition, the requirements outlined for dry machining of composite components further motivates researchers and manufacturers to identify the conditions responsible for temperature rise. Takeshi et al. [26] used three techniques to measure cutting temperature during end milling of CFRP plates. It appeared that the cutting conditions led to exceeding the glass transition temperature of the matrix. Kerrigan and O’Donnell [27] measured both the tool temperature using a wireless embedded thermocouple and the composite part temperature using an infrared thermal imaging camera during edge trimming of CFRP. The obtained results revealed that the steady-state temperature was highly related to the depth. Wang et al. [28] concluded that fiber orientation is a major factor in increasing the cutting temperature during CFRP milling. Sheikh-Ahmad et al. [29] performed temperature analysis during edge trimming of GFRP laminates. The heat flux conducted through the processed surface was shown to be more sensitive to the feed rate rather than the spindle speed.

Nowadays, TCP composite structures have become serious candidates for substituting key components in aircraft. However, their behavior still presents challenging issues at high temperature. This attempt proposes a thermomechanical analysis to highlight the dominating elementary mechanisms and their interactions during fabrication of such materials. Focus was put on heat transfer between phases within TCP composites during dry milling. Especially, analysis was performed on pure polymer matrix behavior. An experimental procedure was rigorously conducted to reveal the influences of fiber orientation and polymer type on temperature to be generated during the milling period. Inspections of fresh surface proved that the control of the mechanisms dominating the chip formation process has great economic impact since it (i) significantly enhances the finish surface integrity of TCP composite structures and (ii) improves tool lifetime by reducing the damage of the abrasive layer.

## 2. Experimental Procedures

### 2.1. Preparation of Polymer Matrix Specimens

The experimental investigation aims to reveal the influence of the matrix type and glass fiber orientation in heat partition and thermal damage effects occurring during machining of TCP composites. The design of experiments includes the type of matrix, fiber orientation, and feed rate as variables.

Epoxy resin from Resoltech Co. (Monaco, France), under the reference 1050 mixed manually with 35% of its volume with the hardener 1055S, was used to prepare pure polymer matrix specimens. The correspondent weight proportion of the two adjuvants of the epoxy matrix was approximated to be 1:3. However, the polyester resin was a reaccelerated cobalt provided by Turkuaz Polyester Co. (Kocaeli, Turkey), under the reference TP280. Table 1 shows the typical characteristics of resins used for preparing composite panels of $300\times 250\times 8\;{\mathrm{mm}}^{3}$.

### 2.2. Preparation of TCP Composite Specimens

The molding process consists first of placing the two phases, namely, the matrix and fibers, in the mold using hand layup technique. Ten layers of unidirectional E-glass cloth of $530\;{g\;m}^{-2}$ from Castro Composites Co. (Pontevedra, Spain) were particularly cut and laid up in the appropriate mold. This laying ensured a fiber volume fraction of approximately 0.3 for the two considered composites. According to the curing cycle, the glass/epoxy panels were kept in the mold for 24 h at room temperature and at a constant pressure level. However, prescriptions imposed a curing cycle of 72 h with a 2% hardener (MEKP) dosage for the glass/polyester panels at room temperature and the same pressure value. The pressure level was reached using dead weight technique. The two composites were prepared in a room environment following supplier instructions, especially as regards humidity level. Table 2 summarizes the details of molding cycles followed for obtaining the two considered composites.

**Table 2:** Curing and cooling cycles for the two considered TCP composites.

Preparation Stage	Parameters	Glass/Epoxy	Glass/Polyester
Layup molding at ambient environment	Time	24 h	72 h +2% MEKP hardener
	Pressure	3 kPa	3 kPa
	Temperature	Room temperature	Room temperature
	Humidity	<70%	<70%
Curing cycle in autoclave	Time	15 h	2 h
	Pressure	3 kPa	3 kPa
	Temperature	60 °C	60 °C
	Cooling time	8 h	2 h

**Figure 1:**
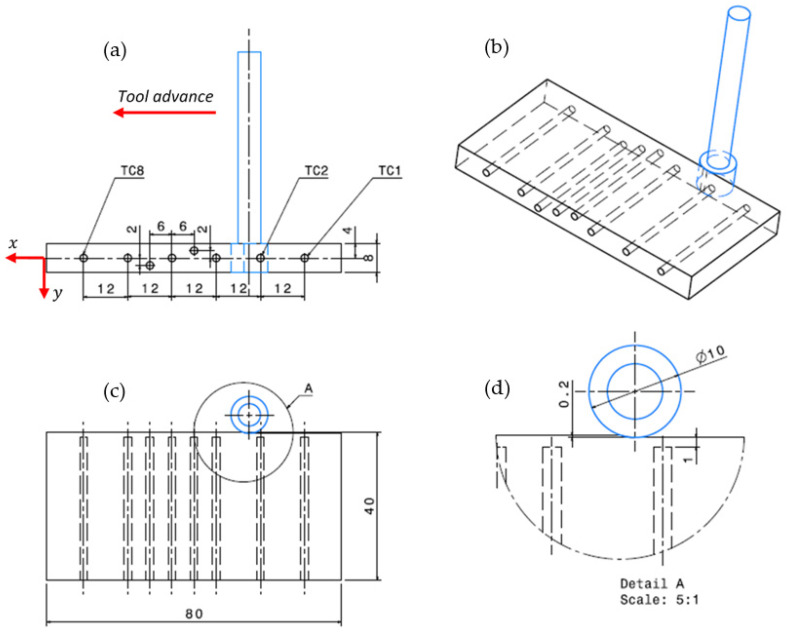
(**a**) Specimen geometry and TC location, (**b**) milling isometry, (**c**) top view of the cutting system, (**d**) enlarged view on the tool–specimen interface.

The composite panels were then postcured in an autoclave at a similar pressure level of 3 kPa and a temperature of 60 °C. The curing cycle in the autoclave was about 15 h for glass/epoxy, while it was about 2 h for glass/polyester. TCP composite panels of 8 min in thickness were finally obtained after mold release and separate cooling periods for the two considered composites. The cooling periods were fixed to prevent deflection of panels while being ejected out from the mold. The composite panels were then laser-cut in an ambient environment to provide specimens of $80\times 40\times 8\;{\mathrm{mm}}^{3}$ (Figure 1), which fit to the milling test requirements.

### 2.3. Milling Test

Milling (i.e., edge trimming) tests were conducted on both pure TCPs and TCP composite specimens using the 5-axis High-Speed CNC Machine Model Spinner U-620 at 12,000 rpm maximum spindle and 19 kW maximum power. As thermal conduction was expected to be sensitive to fiber orientation, milling trials were conducted both parallel (θ=0°) and perpendicular (θ=90°) to the fiber orientation. During processing, the cutting speed, feed rate, and depth of the cut were kept constant at $283{\;m\;\min}^{-1}$ (9000 rpm), $150{\;\mathrm{mm}\;\min}^{-1}$, and 0.2 mm, respectively. All tests were dry and used an abrasive diamond wheel of 60 mm total length. The tool head was 8 mm in height and 10 mm in diameter and consisted of 1 mm effective abrasive layer. The abrasive wheel (diamond grinding points, galvanic metal bonding, reference D126) supplied by Garant Co. ensured excellent cutting quality and optimum service life due to the galvanic nickel binder. The reliability of test series was discussed according to the standard deviation ranges. Figure 1 shows a detailed illustration of the experimental setup, including the tool advance direction, test specimen, and thermocouple location.

### 2.4. Temperature Acquisition

A temperature acquisition system was designed to measure the in-process temperature evolution along the machined surface. A set of eight thermocouples was incorporated into a predrilled blind hole of 2 mm in diameter, which fit the sensor probe diameter. Six of the thermocouples (TCs) were placed at the midthickness of the trim plan. TCs 4 and 6 were intentionally placed at either the top or bottom sides of the specimen to investigate temperature variation at a distance of 1 mm within the subsurface (Figure 1), which was assumed to minimize measurement data loss. The probe tips of all TCs were placed at a low-enough distance (i.e., 1 mm) under the trim plan in order to ensure catching of temperature history at the tool–material interface, particularly when the tool passes in front of the TCs. At the rear thickness side of the specimen, the TCs were glued using an epoxy adhesive in order to prevent the shifting of TC tips from their appropriate locations. The specimen was fixed to the machine jaws to prevent extraction during processing. Because of their appropriate measurement range and reliability, type K TCs provided by Electronic Shop Co. were selected to ensure temperature recording. During tests, the output voltage signal produced by each sensor was directly recorded using an Arduino Mega board connected to the acquisition interface. The data acquisition process was performed via the PLX-DAQ software communicating with an Arduino application for processing the outputs and the data transfer. The temperature history at each TC was recorded at a frequency of 4 Hz. Table 3 summarizes the technical specifications of acquisition system components. The standard deviation was analyzed for each test series to highlight the confidence over the temperature results.

**Table 3:** Technical specification of data acquisition instruments.

Components	Specifications	Range
8 type K TCs (Nickel-chromium/nickel-aluminum)	Measurement range Diameter of conductor Diameter of cable Precision Seebeck coefficient at 0 °C	−270 °C to 1370 °C 0.2 mm 2 mm 2.2% to 0.75% 39.45 μV/°C
8 Max6675 Sensor Modules for Arduino	Voltage Intensity Accuracy Resolution	5 V DC 50 mA ±1.5 °C 0.25 °C
Arduino board based on ATmega2560 with USB connection	Frequency Total inputs/outputs PWM * input Analog input UART **	16 MHz 54 Channels 14 Channels 16 Channels 04 Channels
Dupont male/female connection cables	Appropriate connecting	
Computer with Arduino user interface	Appropriate interface	

* PWM: pulse width modulation. ** UART: universal asynchronous receiver–transmitter.

## 3. Results

### 3.1. Peak Temperature vs. Cutting Length

**Figure 2:**
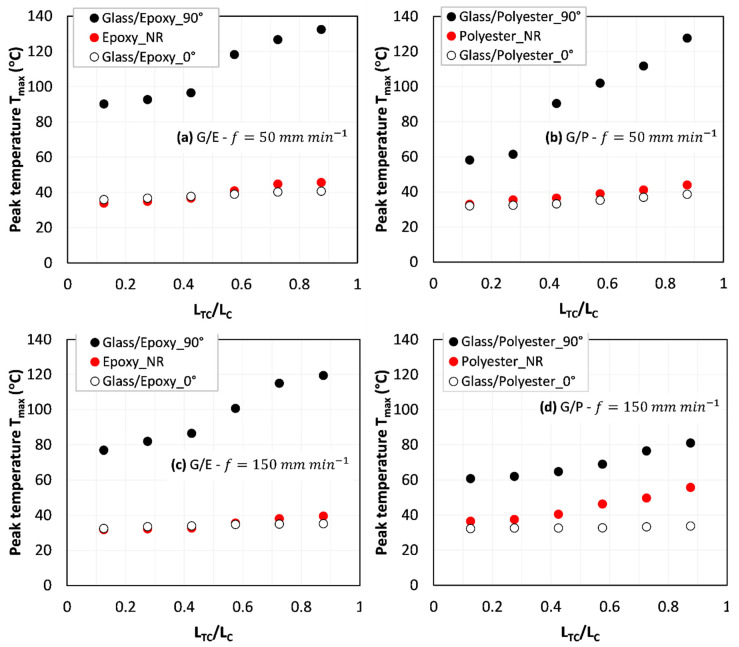
Peak temperature vs. cutting length. (**a**) Glass/epoxy-$f=50{\;\mathrm{mm}\;\min}^{-1}$ (0.09  °C≤SD≤1.6  °C); (**b**) Glass/polyester-$f=50{\;\mathrm{mm}\;\min}^{-1}$ (0.04  °C≤SD≤2.26  °C); (**c**) Glass/epoxy-$f=150{\;\mathrm{mm}\;\min}^{-1}$ (0.04  °C≤SD≤2.58  °C); (**d**) Glass/polyester-$f=150{\;\mathrm{mm}\;\min}^{-1}$ (0.03  °C≤SD≤2.92  °C).

First, outputs of the thermocouples allow plotting the evolution of peak temperature over relative cutting length $\left(\frac{L_{TC}}{L_{C}}\right)$, where *L_TC_* is the distance at which the TC is located by referring to the starting point of cutting, and *L_C_* is the total cutting length. Figure 2 shows the data gathered for both the pure TCP matrices and the TCP composites considered. Since the error bars are too small to be read over the plots, the error ranges were intentionally reported on the title of Figure 2. The standard deviation (SD) intervals inform about the high confidence in the obtained results since the highest SD does not exceed 2.92 °C. This value corresponds to 5.2% of the peak temperature recorded by TC 8 when milling polyester at a $150\;{\mathrm{mm}\;\min}^{-1}\;$ feed. As expected, temperature increases with the tool advance because of cumulative heat flux that is sensitive to the time of contact. When the tool advances, the contact time at interfaces responsible for heat generation increases, and hence, temperature increases. This can be clearly pointed out in the results owing to the two feed rates used. Because the contact period at a $50\;{\mathrm{mm}\;\min}^{-1}$ feed rate is three times higher than at $150\;{\mathrm{mm}\;\min}^{-1}$, the peak temperature exhibits gaps of about 11% for glass/epoxy (Figure 2a,c) and 58% for glass/polyester (Figure 2b,d) when cutting perpendicular to the fiber orientation. However, the reinforcement shows no heat effects when cutting parallel to the fiber. Unexpectedly, glass fibers seem to reduce heat generation even by very small amounts when compared with values obtained from pure polymer matrices. Only results recorded when milling glass/polyester at $150\;{\mathrm{mm}\;\min}^{-1}$ (Figure 2d) shows a remarkable temperature variation about 1.65 times higher than when milling pure polyester.

The findings reveal the typical sensitivity of temperature to the reinforcement when milling perpendicular to the fiber. This was particularly attributed to severe conditions at interfaces between the tool and the cross section of cut the fiber. It should be drawn that transverse sectioning of fibers yields a much larger contact area with a rough surface due to brittle breakage. Domination of pure “mode-II” failure or successive “bending → mode-II” failure in such operation leads to irregular fresh-cut sections of fibers that act as multiple cutters facing the abrasive wheel. Implicitly, this action requires high cutting energy that has to be, totally or mostly, converted to heat. However, when cutting parallel to the fiber, successive “opening mode-I → buckling → mode-II” failure dominates the material removal process. In such a configuration, the interfaces involve mostly the fiber lateral surface and the abrasive wheel. This plays to drastically reduce the fiber-cut surfaces freshly sectioned and favor the reduction of friction, mainly responsible for heat generation. These conclusions coincide well with the data plotted in Figure 2a–c.

The distinguished value of temperature detected when milling glass/polyester at *θ* = 0° (Figure 2d) was partially attributed to the matrix chip that comes incorporated within the interfaces and hence favors the friction generated. SEM inspections conducted on the trim plan of different specimens support this explanation. Adhesion properties at fiber–matrix interfaces can play a great role in the chip formation process and the development of a built-up layer that acts to enhance hard contact conditions with the tool.

### 3.2. Temperature Rate vs. Feed Rate

**Figure 3:**
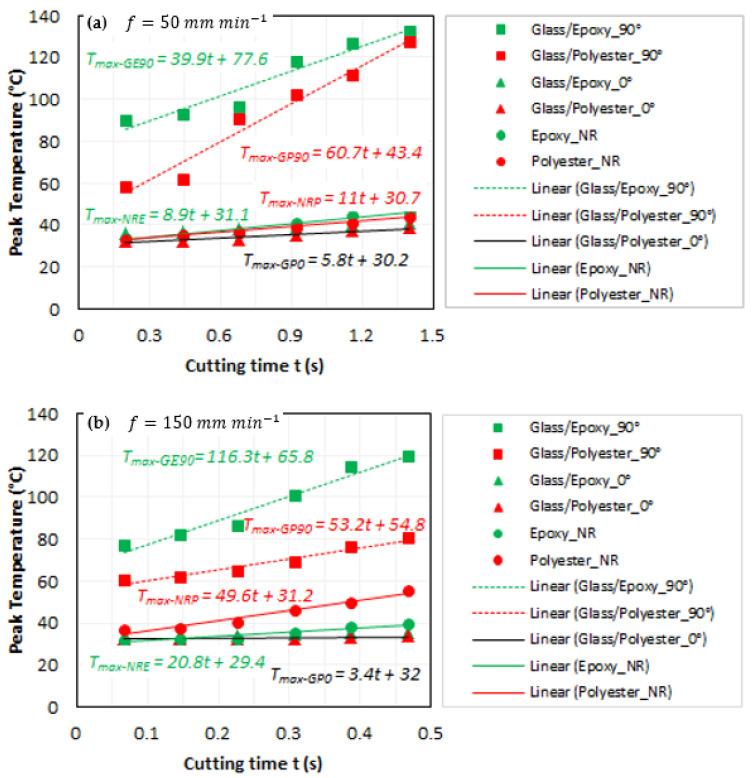
Temperature rate for pure polymer matrices, glass/epoxy and glass/polyester composites: (**a**) $f=50{\;\mathrm{mm}\;\min}^{-1}$; (**b**) $f=150{\;\mathrm{mm}\;\min}^{-1}$.

To further investigate the thermal distribution throughout the specimen, peak temperature was plotted versus cutting time. Figure 3 shows the temperature rate at each of the specimens. The results refer to the same SD intervals mentioned in Figure 2. Linear tendencies were assumed to be reliable enough to reflect the experimental data variation. The temperature evolution can be modeled as:

(1) $$\frac{dT}{dt}=\alpha t+\beta$$

where *α* is a material- and process-dependent constant and *β* is the temperature *T*_0_ at t=0 depending on the initial processing conditions and boundary conditions (i.e., holding of specimen).

Regardless the matrix type, the temperature rate looks increasing as cutting TCP composites parallel to fibers → pure TCP matrices → TCP composites perpendicular to fibers, except in the case of cutting glass/epoxy perpendicular to fibers at the lowest feed rate. Unexpectedly, linear fittings reveal a temperature rate much higher at $150\;{\mathrm{mm}\;\min}^{-1}$ than at $50\;{\mathrm{mm}\;\min}^{-1}$ typically when cutting glass/epoxy perpendicular to the fiber orientation. In fact, this drop in temperature rate of about $65.7\%\;\left(\frac{{{\dot{T}}_{max}^{GE90}|}_{f=150}-{{\dot{T}}_{max}^{GE90}|}_{f=50}}{{{\dot{T}}_{max}^{GE90}|}_{f=150}}\times 100\right)$ observed when the feed rate passes from 150 to $50\;{\mathrm{mm}\;\min}^{-1}$ should involve the matrix transformations because of exceeding the glass transition limit of epoxy. However, it is worth noting that temperature variation remains very close in the two cases, approximately 42 °C.

When cutting parallel to fibers, the experimental findings show linear regressions to be rightly confused irrespective of the matrix type and feed rate. However, it appears that temperatures generated in that case are slightly lower than those recorded during cutting pure polymer matrices. This means that fibers, when arranged parallel to the cutting direction, act as coolers and play a role in reducing heat owing to friction at interfaces. As the thermal conductivity of glass ($\sim 0.02\;{W\;m}^{-1}{\;K}^{-1}$ [30,38]) is known to be much lower than that of epoxy and polyester matrices ($\sim 0.2\;{W\;m}^{-1}{\;K}^{-1}$, [30,31,32,33,34]), fibers seem to act as thermal walls to reduce heat transfer from the interface to the specimen body, which results in relatively low temperature values when cutting parallel to the fibers. In addition, it is assumed that the lateral surface of fibers generates low severe contact and favors sliding of the tool rather than adhesion or abrasion, hence reducing the local friction at tool–composite interfaces.

When cutting perpendicular to the fibers, the rough surface owing to transverse fiber failure enhances friction properties within the interfaces. Frictional effects seem to dominate the relatively low thermal conductivity of fibers, thus resulting in significant temperature levels, which can be neatly observed for the two considered composites.

### 3.3. Milling-Induced Signature into Pure Polymer Matrices

To assess the effects of feed rate on the surface finish, SEM inspections were performed on both rough and fresh-cut surfaces of the considered polymer matrices. The samples were carefully prepared, and micrographs were captured using a scanning electron microscope, TS Quanta 250, at a resolution of 2048 × 1536 pixels, the highest magnification of 600, an accelerating voltage in the range of 12–17 kV, and a low vacuum mode with a constant pressure of 70 Pa.

**Figure 4:**
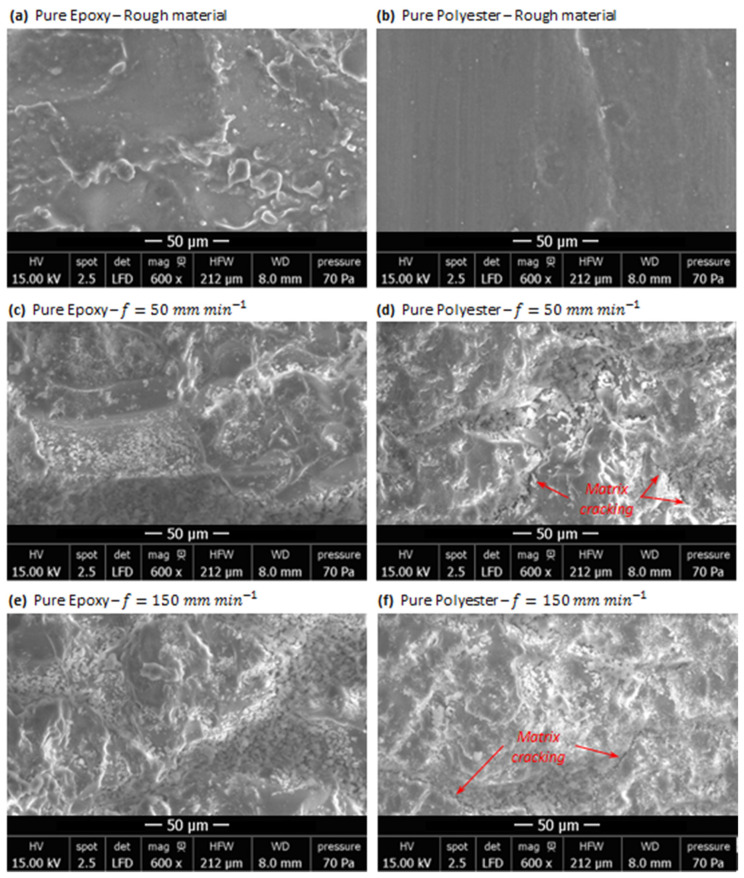
SEM micrographs obtained before and after milling pure polymer matrices.

The inspections revealed the cutting signature of milling pass, resulting in a transition from relatively bidimensional surface topography to tridimensional surface topography. In fact, the fresh-cut areas exhibited a much more irregular topography (Figure 4c–f) than those observed at the initial state irrespective of the matrix type and cutting conditions. However, SEM micrographs showed better surface integrity of epoxy than that of polyester because of the absence of microcracks or failure signatures within the former matrix. Regardless of the cutting conditions, the peak temperature ranged from $\;0.51\;\mathrm{to}\;0.64\;T_{g}$. The minimum gap to glass transition temperature reaching 36% was recorded in milling polyester at $f=150\;{\mathrm{mm}\;\min}^{-1}$.

In contrast, observations reveal the presence of long cracks randomly oriented through the fresh surface of polyester obtained at 50, as well as at $150\;{\mathrm{mm}\;\min}^{-1}$. Without a doubt, the development of such cracks, typically when milling polyester, depends not only on the mechanical properties of the matrix but also on thermal properties. As the thermal conductivities of epoxy and polyester are too close, they should not act to involve significant difference in terms of surface quality owing to generated heat. However, it is worth pointing out the wide gap between specific heat capacity values of epoxy and polyester ($C_{p\text{-}Polyester}\approx 1.46\times C_{p\text{-}Epoxy}$). This lets us rightly assume that specific heat is, in part, responsible for distinguishing the surface integrity state in a potential interaction with the mechanical properties of the two matrices. Crack initiation and development might refer also to residual stresses introduced by milling. Cutting action combined, on the one hand, with local contact pressure owing to abrasive grain edges and, on the other hand, with heat generation plays a role in introducing tension-type residual stresses. This situation motivates surface cracking as a reaction to the two aforementioned mechanisms.

### 3.4. Milling-Induced Signature into TCP Composites

#### 3.4.1. When Cutting Parallel to Fiber

**Figure 5:**
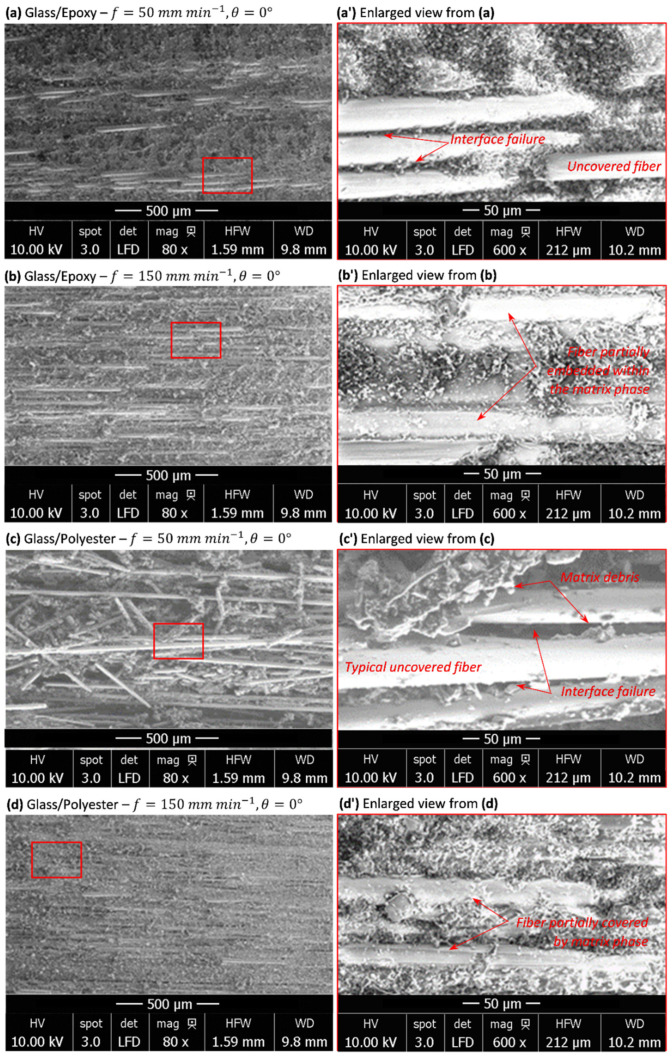
SEM micrographs obtained when milling TCP composites parallel to the fiber orientation. (**a**) Glass/Epoxy-$f=50\;{\mathrm{mm}\;\min}^{-1}$, (**a’**) enlarged view from (**a**,**b**) Glass/Epoxy-$f=150\;{\mathrm{mm}\;\min}^{-1}$, (**b’**) enlarged view from (**b**,**c**) Glass/Polyester-$f=50\;{\mathrm{mm}\;\min}^{-1}$, (**c’**) enlarged view from (**c**,**d**) Glass/Polyester-$f=150\;{\mathrm{mm}\;\min}^{-1}$, (**d’**) enlarged view from (**d**).

Fresh surface analyses were also conducted on unidirectional glass/epoxy and glass/polyester composites. Figure 5a–d show the micrographs of cut area obtained after milling at 50 and $150\;{\mathrm{mm}\;\min}^{-1}$, respectively. Enlarged views (Figure 5a–d) were intentionally presented in order to point out the key mechanisms dominating the material removal process at each of the conducted test.

The microscopic inspections on enlarged views make the estimation of fiber diameter possible. The measurements randomly performed on the above micrographs gave a glass fiber diameter of about 20.5±5.4 μm. The cut land reveals the presence of uncovered fibers embedded in the matrix together with fiber debris forming a separate chip. The fibers embedded within the matrix remain parallel, while the fiber fragments appear to be oriented arbitrarily. The micrograph in Figure 5c exhibits a typical orientation of released fiber fragments. Once the chip is being separated from the specimen, the wheel acts to eject the released material by inertial effects. The dusty nature of the separated chip, involving fiber fragments alone or covered with the matrix resin, results in a series of fiber fracture through the effect of multiple cut edges of abrasive grains on the wheel. When milling parameters change, the material removal process seems to switch from tool–material interfaces to fiber–matrix interfaces.

- At $50\;{\mathrm{mm}\;\min}^{-1}$ (Figure 5a,c), the material removal process looks different from that at the highest considered feed irrespective of the matrix type. In this case, the rest of the fibers embedded in the matrix and the free fiber fragments appear completely uncovered in the matrix phase. Observations of the respective enlarged views (Figure 5a,c) prove the total failure of fiber–matrix interfaces with the presence of pure polymer matrix debris. In fact, as glass fiber is of very low thermal conductivity, it plays a role in locally stopping heat transfer from the matrix and thus acts to localize temperature at its lateral surface (i.e., interface), which significantly enhances property degradation at fiber–matrix interfaces and leads to premature failure. On the other hand, one can recall that the specific heat capacity of glass ($835\text{–}840\;J\;{\mathrm{kg}}^{-1}\;K^{-1}$ [38,39]) is significantly lower than those of the considered matrices. This entails that at a specific heat threshold, glass stops to absorb heat while the matrix absorbs much higher quantity. These effects will localize heat within the fiber–matrix interfaces, which have been found to be forced to resist the opposite flux, yielded from either phase. This will accelerate fiber–matrix failure. Such failure mechanisms might also be observed through the long uncovered rests of fibers that are still embedded on the cut surface. The same failure mechanisms dominate the material removal process of both glass/epoxy and glass/polyester. However, experimental measurements (see appropriate plots in Figure 3) confirm the same temperature level when milling parallel to the fiber whatever the composite type is. This entails that in such cutting configuration, the chip formation stage is highly governed by the fiber phase, while the matrix phase fails prematurely, long before, hence favoring direct contact between the wheel abrasive grains and the glass fiber units.
- At $150\;{\mathrm{mm}\;\min}^{-1}$ (Figure 5b,d), the temperature rate is much lower than that obtained at $50\;{\mathrm{mm}\;\min}^{-1}$ $\left(\frac{{\dot{T}|}_{f=150}}{{T|}_{f=50}}=0.58\right)$. This lets us consider that heat localizes much less when feed increases. Referring to micrographs of Figure 5b,c, it can be seen that the fiber–matrix interface exists, and no matrix loss around the fiber units was observed out of the trim plan. Over the fresh surface, the fibers look covered or partially embedded by the matrix phase along either visible side. Temperature seems not to reach a value high enough to yield interface loss. In such case, a separated chip forms fragments consisting of glass fiber covered with the surrounding resin. Hence, mechanical damage rather than heat localization effects mainly governs the material removal process. While interface loss owing to heat localization at $50\;{\mathrm{mm}\;\min}^{-1}$ involves discontinuities in heat transfer between composite phases, continuity seems to be ensured at $150\;{\mathrm{mm}\;\min}^{-1}$, but heat effects were found to be attenuated compared with mechanical ones.

#### 3.4.2. When Cutting Perpendicular to Fiber

**Figure 6:**
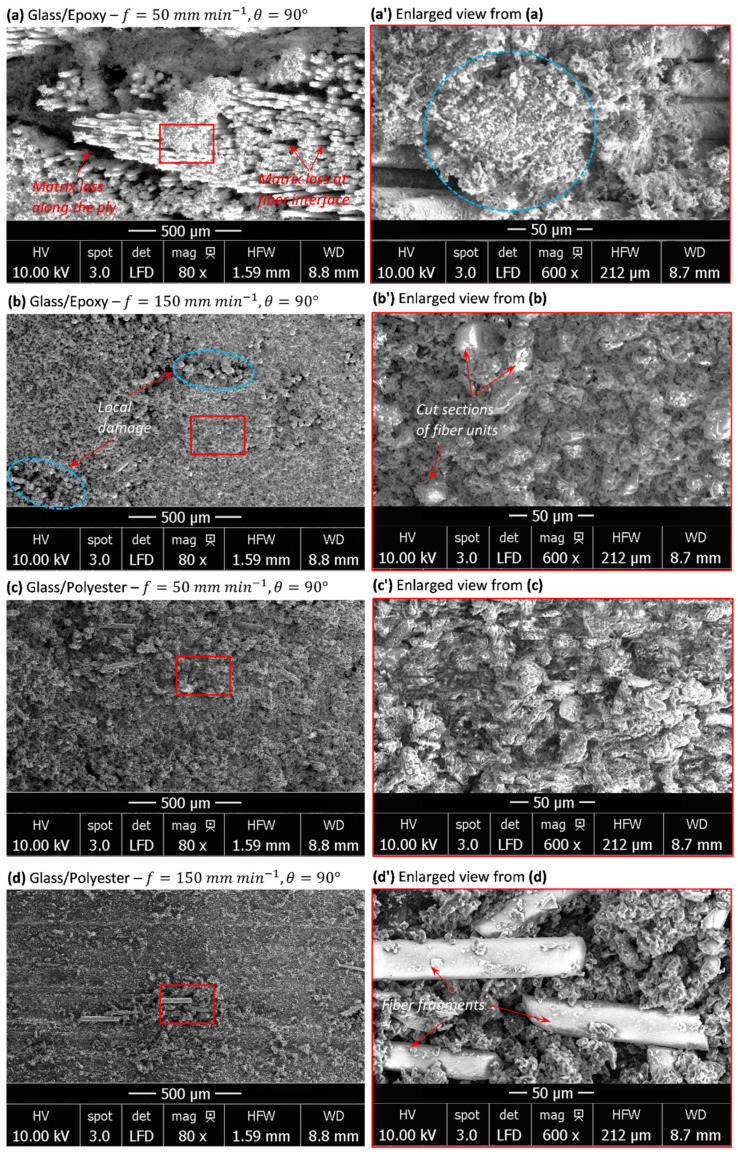
SEM micrographs obtained when milling TCP composites perpendicular to the fiber orientation. (**a**) Glass/Epoxy-$f=50\;{\mathrm{mm}\;\min}^{-1}$, (**a’**) enlarged view from (**a**,**b**) Glass/Epoxy-$f=150\;{\mathrm{mm}\;\min}^{-1}$, (**b’**) enlarged view from (**b**,**c**) Glass/Polyester-$f=50\;{\mathrm{mm}\;\min}^{-1}$, (**c’**) enlarged view from (**c**,**d**) Glass/Polyester-$f=150\;{\mathrm{mm}\;\min}^{-1}$, (**d’**) enlarged view from (**d**).

In order to investigate the sensitivity of the heat signature to material structure, SEM inspections were conducted on specimens milled perpendicular to the fiber orientation. Figure 6 illustrates micrographs of fresh surfaces obtained at the considered feeds and matrices. From the inspections, the difference between cut surface aspects generated on glass/epoxy and glass/polyester was pointed out. The following observations can be especially drawn:

- For glass/epoxy, inspections reveal catastrophic failure, typically at $50\;{\mathrm{mm}\;\min}^{-1}$. Matrix loss takes place within plies and surrounding fiber units yielding deep damage on the trim plan (Figure 6a). Cut fibers exhibit neat mode-II failure throughout the milled land. Matrix cracking seems to result from combined effects, mechanical and thermal, providing that temperature histories (Figure 3a) implicitly inform about heat generation. In fact, the highest heat localization should particularly occur when milling perpendicular to the fibers, and furthermore, for glass/epoxy, since the peak temperature reaches the maximum values at that configuration. While no fiber fragment was observed on the cut surface, free matrix residues potentially released from interfaces were detected at the trim plan (Figure 6a). Provided a fixed depth of the cut (i.e., 0.2 mm), the material removal process involves a series of neat fractures of successive fiber plies, hence resulting in a dusty chip with a relatively uniform chip size. In contrast, when cutting parallel to the fiber, the chip forms are irregular, consisting of relatively long fiber fragments (see the typical chip size in Figure 5c). However, at $150\;{\mathrm{mm}\;\min}^{-1}$, micrographs exhibit a smoother cut area compared with the former one. Despite the presence of local degradation by the effect of matrix cracking (Figure 6b), subsurface damage appears less critical than that developed at a low feed rate.
- For glass/polyester, fresh surfaces inform about the cutting process much less severe than that observed in glass/epoxy despite insignificant difference in results existing when the feed rate varies. As regards glass/epoxy, matrix loss is also more visible at $50\;{\mathrm{mm}\;\min}^{-1}$ (Figure 6c,d). The temperature drop pointed out when the feed decreases should play a key role in the evolution of damage mechanisms. Because of the effect of relatively low thermal conductivity, glass fibers act as thermal walls to limit heat transfer. Heat localizes within the intermediate matrix phase and leads to properties’ degradation. This decides on the chip formation mechanisms and governs the surface integrity at the trim plan. Regardless of the cutting feed, the matrix phase seems to relatively dominate the initial stage of the material removal process. Before fiber failure, the matrix should release the surrounding fiber, hence motivating the uncovered fiber to be cut during wheel advance.

## 4. Discussion

In order to understand heat distribution through the cut material, temperature history was also recorded through the specimen thickness b. Outputs issued from TCs 4, 5, and 6 located on the top side (y/b=−0.25), within the midplan (y/b=0), and on the bottom side (y/b=0.25), respectively, are summarized in Table 4.

Basically, it was found that temperature values on the top side ($T_{TC4}$) are neatly lower that those ($T_{TC6}$) depicted on the bottom side. As regards the midplan temperature $\left(T_{TC5}\right)$, most gaps $\left(\frac{T_{TC4}-T_{TC6}}{T_{TC5}}\times 100\right)$ were especially recorded while cutting glass/epoxy perpendicular to the fiber and reach 22% at $50\;{\mathrm{mm}\;\min}^{-1}$ and 16.6% at $150\;{\mathrm{mm}\;\min}^{-1}$. Such a difference is mainly attributed to the boundary conditions, resulting in the clamping of the specimen on the machine [40]. In fact, as the top surface of the specimen is in contact with only ambient air, it favors temperature evacuation and thus plays a role in reducing heat localization at a subsurface. However, the bottom surface is close enough to the steel table of the milling machine, which potentially involves incident heat flux against the bottom surface, hence preventing rapid heat transfer with the cutting environment.

Outputs obtained during cutting of TCP composites parallel to the fibers and pure polymer matrices did not lead to significant disparate temperatures with relatively low error. As regards pure polymer matrices, the standard deviation did not exceed 1.62 °C (TC 6), which corresponds to $\sim 0.0379{\times T_{max}^{P}|}_{50\mathrm{mm}/\min}$, reached in polyester. However, when milling TCP composites parallel and perpendicular to the fiber, the highest standard deviations were also recorded both at TC 6 and equal to $0.96\;{}^{\circ}C\;\sim \;0.0266\times {T_{max}^{G/P}|}_{50\mathrm{mm}/\min}$ and $2.48\;{}^{\circ}C\;\sim \;0.0225\times {T_{max}^{G/E}|}_{150\mathrm{mm}/\min}$, respectively. These relatively low discrepancies prove the high reliability of the experimental measurement procedure and reflect the high efficiency of the discussed data.

## 5. Conclusions

TCP composite structures have become increasingly popular in aerospace industries since the last decades. However, the control of their processing remains understudied. Thermomechanical coupling seems to sensitively decide on the obtained parts. Based on the analysis of thermomechanical signature owing to dry milling of such materials, the following remarks can be drawn:

- Milling TCP composites parallel to the fiber weakly affects the temperature history when compared with that obtained in pure polymer matrices. However, the peak temperature was found to be slightly more sensitive and typically inversely proportional to the feed rate. The lateral surface of the fiber coming into contact with the tool plays a role in favoring sliding at wheel–fiber interfaces, hence preventing severe friction, considered as the main source for generating heat. SEM inspections highlight elementary mechanisms dominating the material removal process. At $50\;{\mathrm{mm}\;\min}^{-1}$, quasi-total loss of resin surrounding the fiber units precedes the final stage of chip formation due to the very low specific heat and conductivity of glass, which plays a role in localizing heat at interfaces and, hence, favors its degradation. At $150\;{\mathrm{mm}\;\min}^{-1}$, the temperature rate was found to be significantly lower and heat effects appeared to be attenuated compared with the mechanical ones.
- In contrast, measurements show a significant increase in peak temperature when milling composites perpendicular to the fiber by referring to it, resulting in milling pure polymer matrices. The transverse brittle failure of fiber units leads to a fresh area with an irregular aspect rough enough to drastically enhance friction. The severe contact conditions yield heat flux capable of switching peak temperature by a large gap when compared with that found when milling parallel to the fiber. Typically, a catastrophic failure resulting in matrix loss takes place within plies and the surrounding fiber units, yielding deep damage on the trim plan when milling glass/epoxy at $50\;{\mathrm{mm}\;\min}^{-1}$. A chip forms because of a series of neat fractures, leading to a uniform chip size. However, subsurface damage appears to be less critical at $150\;{\mathrm{mm}\;\min}^{-1}$ in spite of local degradation, resulting in matrix cracking.
- Chip formation is mainly governed by the fiber phase, while the matrix phase fails prematurely, long before, when milling parallel to the fiber at relatively low feed rates. However, the matrix phase seems to dominate the initial stage of the material removal process when milling perpendicular to the fiber, regardless of the cutting feed.
- It was revealed that TCP composites’ integrity sensitively depends on cutting conditions. The temperature generation on a fresh surface switches with the fiber orientation, hence affecting the behavior of the final structure and tool lifetime. This should motivate us to select appropriate parameters for better control of the economic impact on the glass-fiber-reinforced TCP structures.

## Figures and Tables

**Table 1:** Properties of epoxy and polyester resins used for preparing the specimens.

Properties	Epoxy 1050	Polyester TP 280
Appearance	Opalescent neutral liquid	Clear blue
Density ρ	$1.27\pm 0.05{\;g\;\mathrm{cm}}^{-3}$	$1.10\pm 0.05{\;g\;\mathrm{cm}}^{-3}$
Glass transition temperature *T_g_*	77 °C	87 °C
Thermal conductivity λ at 20 °C	$0.20\text{–}0.22{\;W\;m}^{-1}{\;K}^{-1}$ [30,31,32]	$0.18{\;W\;m}^{-1}{\;K}^{-1}$ [33,34]
Specific heat capacity *C_p_*	$1300{\;J\;\mathrm{kg}}^{-1}{\;K}^{-1}$ [31,35]	$1870\text{–}1930{\;J\;\mathrm{kg}}^{-1}{\;K}^{-1}$ [36,37]
Dry extract in volume	33 ± 2%	31 ± 1%
Viscosity κ	1.043 Pa· s	0.647 Pa· s
Hardener type	1055S	MEKP
Gel time	210 min at 23 °C with 1055S hardener	30 (at 25 °C with 2% of MEKP hardener)

**Table 4:** Peak temperature (°C) vs. relative thickness recorded at TCs 4, 5, and 6.

$Feed\;\left({\mathrm{mm}\;\min}^{-1}\right)$	$b_{TC}/b$	Epoxy NR	G/E 0°	G/E 90°	Polyester NR	G/P 0°	G/P 90°
50	−0.25	39.25 ± 0.49	38.5 ± 0.28	99 ± 1.08	37.5 ± 0.56	34 ± 0.10	100.75 ± 0.81
	0	41 ± 0.42	39 ± 0.27	118.25 ± 1.29	39 ± 1.12	35.25 ± 0.42	102 ± 0.65
	0.25	42 ± 0.73	39.5 ± 0.14	125 ± 0.75	42.75 ± 1.62	36 ± 0.96	105.25 ± 1.61
150	−0.25	34.75 ± 0.43	34.25 ± 0.09	93.5 ± 1.42	42.25 ± 0.75	32.75 ± 0.05	66.25 ± 1.11
	0	35.5 ± 0.33	34.75 ± 0.17	100.75 ± 1.72	46.25 ± 1.15	32.75 ± 0.15	69 ± 0.96
	0.25	37 ± 0.50	34.5 ± 0.19	110.25 ± 2.48	48.75 ± 0.66	33 ± 0.08	69.5 ± 0.39

## Data Availability

Not applicable.
